# Boron-Enhanced Mitochondrial Repair: DeepA-I Tissue Regeneration

**DOI:** 10.1155/sci5/5343930

**Published:** 2025-10-26

**Authors:** Beyza Yılmaz, Basak Dalbayrak, Mustafa Kucukali, Pinar Uysal Onganer, Elif Damla Arısan

**Affiliations:** ^1^Institute of Biotechnology, Gebze Technical University, Gebze, Kocaeli, Türkiye; ^2^Pyrosoft Inc. Gebze Technical University Technopark, Gebze, Kocaeli, Türkiye; ^3^Cancer Mechanisms and Biomarkers Research Group, School of Life Sciences, College of Liberal Arts and Sciences, University of Westminster, London, UK; ^4^Original Bio-Economy Resources Center of Excellence (OBEK), Gebze, Kocaeli, Türkiye

**Keywords:** Boron supplementation, oxidative stress, reactive oxygen species (ROS), tissue repair

## Abstract

Cellular metabolism is a key regulator of tissue repair and regeneration, with mitochondrial function playing a central role in energy production and cellular homeostasis. Dysfunctional mitochondria, often due to excessive reactive oxygen species (ROS), contribute to oxidative stress, impaired wound healing, and chronic inflammation. This study investigates the therapeutic potential of DeepA-I, a Boron-enriched compound, in enhancing mitochondrial health, reducing oxidative damage, and promoting cellular repair in human umbilical vein endothelial cells (HUVEC) and mouse embryonic fibroblasts (MEF). Boron quantification via inductively coupled plasma optical emission spectroscopy (ICP-OES) confirmed its presence in DeepA-I. Cytotoxicity assessment (MTT assay) demonstrated its safety, while fluorescence microscopy (DAPI, MitoSPY, DCFDA) revealed reduced ROS levels and preserved mitochondrial integrity. A scratch assay showed accelerated cell migration and wound closure in DeepA-I-treated cells. Western blot analysis indicated the downregulation of Akt (a proliferation marker) and the upregulation of NRF2, a key regulator of oxidative stress resistance, without affecting apoptosis-related proteins. These results suggest that DeepA-I, via its Boron-mediated mechanisms, enhances mitochondrial function, mitigates ROS-induced damage, and improves tissue repair, positioning it as a promising therapeutic candidate for inflammatory and degenerative conditions.

## 1. Introduction

Cellular homeostasis is essential for the survival and function of all living organisms, relying on a finely tuned balance between energy production, metabolic regulation, and signalling pathways mediated by cytokines, growth factors, and hormones. Reactive oxygen species (ROS), which are naturally produced byproducts of aerobic metabolism, are critical modulators of redox balance and metabolic processes [1]. However, when ROS production exceeds cellular antioxidant capacity, this balance is disrupted, resulting in oxidative stress, chronic inflammation, and impaired cellular function—hallmarks of tissue damage and delayed wound healing [2]. The wound healing process is susceptible to redox imbalances. Elevated ROS levels can degrade extracellular matrix (ECM) proteins, impair the activity of fibroblasts and keratinocytes, and perpetuate inflammatory signalling, collectively hindering tissue repair [3]. Paradoxically, ROS are also essential mediators of wound healing, playing pivotal roles in cell proliferation, inflammation resolution, and ECM remodelling [4]. This dual role highlights the importance of precise redox regulation in harnessing the beneficial effects of ROS while minimising their detrimental consequences. Accordingly, maintaining ROS homeostasis is crucial for effective tissue regeneration, and therapeutic strategies that restore redox balance hold significant promise for improving wound healing outcomes.

Micronutrients play a vital role in regulating cellular redox status and metabolic networks. Among these, the trace element Boron has emerged as a key modulator of metabolic and redox processes, exhibiting a unique dual functionality [5, 6]. Boron is involved in essential cellular activities, including energy metabolism, enzymatic regulation, and inflammatory control [4, 5]. It stabilises cellular membranes, supporting structural integrity across diverse biological systems [5]. Its therapeutic potential is supported by growing evidence of its ability to accelerate wound healing, enhance the detoxification of heavy metals, and upregulate endogenous antioxidant defenses [5, 7]. Notably, Boron's capacity to mitigate oxidative damage while supporting physiological processes—including bone metabolism, mineral absorption, and immune function—positions it as a promising agent for managing oxidative stress-related pathologies and promoting tissue repair [7].

At the molecular level, Boron has been shown to form a complex with 6-phosphogluconate, thereby inhibiting the activity of 6-phosphogluconate dehydrogenase (G6PD) in the pentose phosphate pathway [8]. This inhibition reduces NADPH production, limiting the availability of a key pro-oxidant cofactor in ROS generation [5]. Beyond metabolic regulation, Boron exhibits direct antioxidant effects by enhancing intracellular antioxidant reserves, scavenging ROS, and potentially activating antioxidant enzymes [9–12]. These multifaceted mechanisms enable Boron to confer broad cellular protection against oxidative stress-induced damage, including DNA strand breaks and lipid peroxidation.

Significantly, the antioxidant activity of Boron is complemented by its ability to modulate tissue repair processes, mainly through the regulation of cell migration and ECM dynamics [13, 14]. Effective wound healing requires the coordinated migration of fibroblasts and keratinocytes, ECM remodelling, and growth factor signalling [15]. Boron has been shown to regulate key ECM-remodelling enzymes, including elastase, trypsin-like proteases, collagenase, and alkaline phosphatase, in fibroblasts, thereby promoting a microenvironment favourable for cell migration and tissue regeneration [16, 17]. In vitro studies confirm that Boron facilitates ECM transformation and fibroblast migration, while clinical observations report enhanced healing in Boron-based burn wound treatments [18]. This convergence of redox regulation and ECM remodelling underscores Boron's unique therapeutic potential in addressing oxidative stress and impaired tissue repair.

Building on these mechanistic insights, DeepA-I, a Boron-enriched supplement developed by Pyrosoft, offers a targeted intervention to enhance wound healing and cellular metabolic function. Boron's multifaceted biological roles are well established, including antioxidant activity, metabolic regulation, and support for tissue repair. Boron-supplemented DeepA-I presents a promising and encouraging therapeutic strategy. In this study, we investigate the effects of DeepA-I on key aspects of epithelial cell function, specifically examining its impact on mitochondrial activity, ROS homeostasis, cell viability, and migration, to elucidate its potential to promote tissue regeneration.

## 2. Materials and Methods

### 2.1. Cell Culture

Human umbilical vein endothelial cell (HUVEC) (ATCC, CRL1730) and mouse embryonic fibroblast (MEF) (ATCC, C57BL/6) cell lines were incubated with Dulbecco's Modified Eagle Medium (DMEM) supplemented with 10% FBS and 1% penicillin/streptomycin in a 5% CO_2_ incubator at 37°C (growth condition). When the cellular confluency reached 80%, the cells were detached using trypsin and counted with the QuantaCELL (Optofil) before being centrifuged at 1500 rpm for 5 min. The appropriate cellular medium was used, and cells were seeded as specified by the experimental design.

### 2.2. Elemental Analysis

For the elemental analysis, after the DeepA-I solution, which was mainly a NaCl solution electrolyzed, was supplemented with nitric acid, the following elements were detected: Boron, calcium, potassium, magnesium, sodium, and sulfur. The mineral content of the DeepA-I was determined by inductively coupled plasma optical emission spectrometry (ICP-OES, Agilent, CA, USA).

### 2.3. Cell Viability-MTT Assay

The MTT (3-(4,5-dimethylthiazol-2-yl)-2,5-diphenyltetrazolium bromide) assay was conducted to determine the cell viability after different concentrations of DeepA-I solutions (diluted with DMEM, 1:10, 1:100, 1:1000, 1:5000, and 1:10,000) applications for 24 h to HUVEC and MEF cell lines. For the MTT assay, 5 × 10^3^ cells/well were seeded on a 96-well plate, and after cellular attachment was achieved, diluted DeepA-I solutions were applied for 24 h under growth conditions. After 24 h of incubation with the DeepA-I solution, 10 μL of MTT reagent was added for 4 h. Then, the formazan crystals were dissolved in dimethyl sulfoxide (DMSO) and read at 570 nm using a BioTek MicroPlate Reader 800 (US) [19].

### 2.4. Fluorescence Images

3 × 10^4^ cells/well were seeded in a 24-well plate for fluorescent staining. The DeepA-I solution was diluted to the same concentrations as the MTT assay and applied 24 h after cell attachment was provided. At the end of the incubation, the existing medium was replaced with PBS containing fluorescent dyes (final concentrations: 1 μM MitoSPY, 0.5 μg/mL DAPI, and 0.5 μM DCFDA), and the sample was then incubated for 15 min before imaging with a fluorescence microscope at the appropriate wavelengths.

### 2.5. Wound Healing Assay

For the wound healing experiment performed on HUVEC and MEF cells, cells were seeded in 24-well plates at a density of 3 × 10^5^ cells per well. After 24 h, the bottom of each well containing the cells was scratched using a sterile 10 μL pipette tip, and cell residues were removed with the medium. The cells were then washed with PBS. Then, the DeepA-I solution was applied to the cells at concentrations of 1:10 and 1:1000. The scratches were imaged daily in the same region, and the analysis was performed using the ratio of each day's control.

### 2.6. Western Blotting

Cells were collected after 24 h treatment with DeepA-I at 1:10 and 1:1000. For protein isolation, the cell pellet was washed with 1xPBS. Then, the pellet was dissolved in M-PER (Mammalian Protein Extraction Reagent) solution, which included phosphatase and protease inhibitors, and kept on ice for 20 min. After incubation, centrifugation was performed at 13,200 rpm for 20 min at 4°C. The supernatants were collected and measured via Bradford assay at 595 nm to determine the concentrations. Equal concentrations of proteins (30 μg) were loaded with 4X Laemmli Sample buffer and run on sodium dodecyl sulfate-polyacrylamide gel electrophoresis (SDS-PAGE). Then, the protein samples were transferred from the gel to the polyvinylidene difluoride (PVDF) membrane with semi-dry transfer. After the transfer process, membranes were blocked with 5% milk powder prepared in TBS-T. The membrane was incubated with an appropriate primary antibody overnight at 4°C. The membrane was washed with TBS-T and TBS and then incubated with HRP-linked secondary antibodies. After the second incubation, the membrane was rewashed, treated with ECL, and imaged on the ChemiDoc, Bio-Rad, US. For the Western blotting experiment, β-actin (#4967), AKT (#9272), Caspase-3 (#9663), Nrf2 (#20733), PARP (#9532), and HRP-linked secondary antibody (#7074) were purchased from Cell Signalling Technology.

### 2.7. Data Analysis

The statistical analysis for biological replicates in all experiments was performed using two-way ANOVA with GraphPad Prism version 9.2.0, GraphPad Software, Boston, Massachusetts, USA, https://www.graphpad.com.

## 3. Results

The elemental analysis showed that the sodium concentration in the DeepA-I solution was approximately 1408 mg/kg, and the solution was supplemented with Boron at a concentration of 111 mg/kg. Additionally, DeepA-I has been found to contain trace amounts of calcium, potassium, magnesium, and sulfur (Table 1).

**Figure 1:**
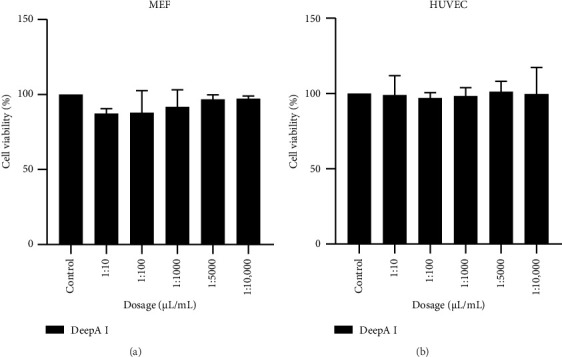
Dose-dependent MTT results for DeepA-I in MEF (a) and HUVEC (b) cells. Error bars indicate standard deviations of 3 biological and 4 technical replicates. For control groups, cells were treated with a medium without DeepA-I. ns. *p* > 0.05.

MTT results were then analyzed using GraphPad 9.2.0 with a two-way ANOVA and Tukey's multiple comparisons test. After MTT cytotoxicity analysis, the results show that the DeepA-I solution is nontoxic in MEF (Figure 1(a)) and HUVEC cells (Figure 1(b)).

**Figure 2:**
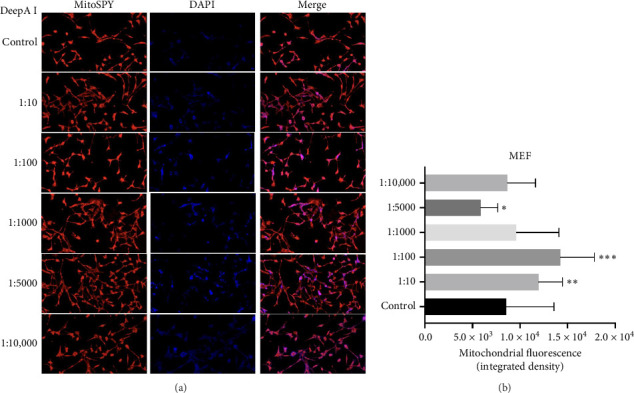
(a) Representation of mitochondria (MitoSPY, red) and cell nuclei (DAPI, blue) after 24 h of DeepA-I treatment (1:10, 1:100, 1:1000, 1:5000, and 1:10,000) in MEF cells. The representative image is selected from 2 repetitive images. The scale bar is 150 μm. (b) Quantitative analysis of mitochondrial fluorescence under control and DeepA-I-treated conditions. Data are represented as mean ± SEM (^∗∗∗^*p* < 0.001, ^∗∗^*p* < 0.01, ^∗^*p* < 0.05).

The cells were exposed to DeepA-I in a dose-dependent manner to evaluate the mitochondrial function of the MEF and HUVEC cells. In MEF cells, compared to the control group, all Deep A-I concentrations tested, except 1:5000 caused an increase in mitochondrial fluorescence, with the most pronounced effects observed at 1:100 and 1:10 dilutions. These results suggest that Deep A-I increases mitochondrial content or activity in MEF cells in a dose-dependent manner (Figure 2).

**Figure 3:**
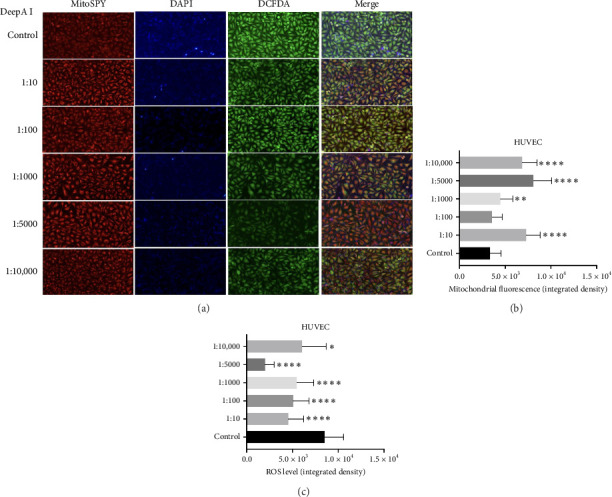
(a) Representative images of mitochondria (MitoSPY, red), cell nuclei (DAPI, blue), and ROS activity (DCFDA) following DeepA-I treatment 24 h (1:10, 1:100, 1:1000, 1:5000, and 1:10,000) in HUVEC cells. The representative image is selected from 2 repetitive images. The scale bar is 150 μm. (b) Quantitative analysis of mitochondrial fluorescence under control and DeepA-I-treated conditions. (c) Quantitative analysis of ROS level under control and DeepA-I-treated conditions. Data are represented as mean ± SEM (^∗∗∗∗^*p* < 0.0001, ^∗∗^*p* < 0.01, ^∗^*p* < 0.05).

HUVEC cells were also stained with the ROS indicator dye DCFDA after treatment with increasing concentrations of DeepA-I (Figure 3). The mitochondrial fluorescence intensity, indicated by MitoSPY staining, increased with Deep A-I treatment across all tested dilutions (1:10, 1:100, 1:1000, 1:5000, 1:10,000), suggesting enhanced mitochondrial activity or biogenesis (Figure 3(b)). Concurrently, ROS levels, as measured by DCFDA staining, decreased significantly across all dosages of Deep A-I, indicating a reduction in oxidative stress within the cells (Figure 3(c)).

**Figure 4:**
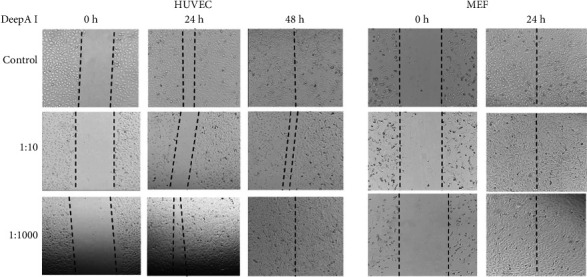
Wound healing-migration analysis was performed in HUVEC and MEF cells following two DeepA-I treatments. Microscopic images of HUVEC cell migration were taken at 0, 24, and 48 h. The same analysis was done at only 24 h exposure of MEF cells. Wound closure (%) in both cell lines was analysed to track the migration profile of the cells in the presence or absence of DeepA-I. Representative images were selected from 2 biological replicates.

To evaluate the effect of the Boron-enhanced DeepA-I effect on wound-healing scratch, an assay was performed to analyze migration in both HUVEC and MEF cell lines. The motility and migration of both cell lines were imaged at time points of 0, 24, and 48 h, respectively. Microscopic images obtained from HUVEC cells using migration analysis showed that control cells and cells treated with DeepA-I at a ratio of 1:1000 closed the scratch within 48 h. In contrast, cells treated with DeepA-I at a 1:10 ratio did not close entirely within 48 h (Figure 4; Supporting Figure 1). As shown in Figure 4, the wound closure (%) was 100% in MEF cell lines in both concentrations of DeepA-I treatment for 24 h. Thus, we concluded that DeepA-I treatment at two concentrations induced wound closure due to increased migration.

**Figure 5:**
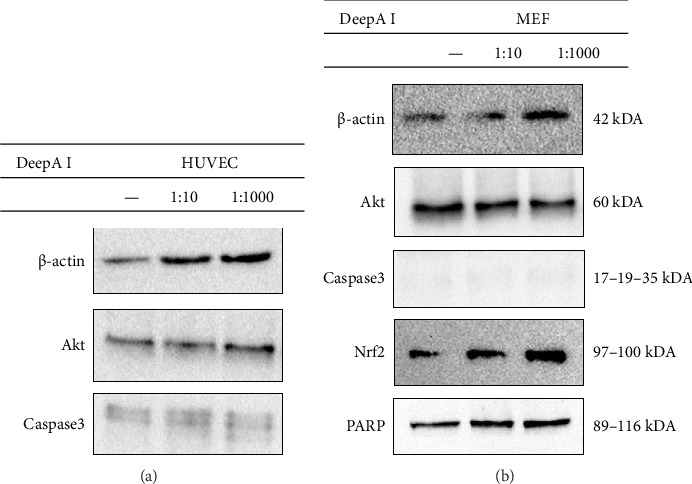
Analysis of the changes in protein expression by immunoblotting. Cell survival, antioxidant capacity, apoptosis, and autophagy pathways were analyzed at the protein level in HUVEC (a) and MEF (b) cells with and without DeepA-I treatment. β-actin was used as a control. Representative images were selected from 2 biological replicates.

To assess the potential role of DeepA-I on cell survival, proliferation, and death, the DeepA-I solution was applied at concentrations of 1:10 and 1:1000 for 24 h on both HUVEC and MEF cells (Figure 5). The results showed that the Akt level, playing a role in cell survival, increased compared to the control when DeepA-I was applied at a ratio of 1:1000 in HUVEC cells. Moreover, according to immunoblotting analysis, the level of Caspase-3 increased in the cells treated with DeepA-I at a concentration of 1:10 compared to the control. At the same time, there was no significant change in the cells treated with a concentration of 1:1000 (Figure 5(a)).

Additionally, the results show that the Akt level decreased and NRF2, which plays a role in the development of cellular resistance to oxidants, increased compared to the control in MEF cells applied with DeepA-I at the ratios of 1:10 and 1:1000. Additionally, downregulation of Caspase-3, a regulator of apoptosis, was detected in MEF cells. There was no significant change in these levels with the application of DeepA-I. Additionally, it was observed that the levels of PARP, which play a critical role in DNA repair, increased in MEF cells treated with DeepA-I at ratios of 1:10 and 1:1000 (Figure 5(b)).

## 4. Discussion

In this study, we investigated the effects of DeepA-I, a novel Boron-enriched supplement, on maintaining redox signalling, cellular metabolism, and redox homeostasis, as well as wound healing-related processes in epithelial (MEF) and endothelial (HUVEC) cells. Emerging evidence highlights the pivotal role of Boron in modulating oxidative stress, cellular metabolism, and tissue repair, positioning it as a promising therapeutic micronutrient. Specifically, Boron has been shown to exert antioxidant effects by reducing ROS levels, thereby maintaining cellular homeostasis and protecting against oxidative damage [10–12].

Our data support this trend, demonstrating that DeepA-I supplementation did not exert cytotoxic effects on either epithelial or endothelial cells, even at relatively high Boron concentrations (Table 1 and Figure 1). This result aligns with previous reports [20, 21] suggesting that Boron supplementation within physiologically tolerable ranges is safe and well-tolerated by various cell types.

We have demonstrated that DeepA-I treatment reduced intracellular ROS levels in HUVEC cells (Figure 3). This antioxidant effect was observed across all tested doses in HUVEC or MEF cells (Figures 2 and 3). Our data suggest that DeepA-I mitigates oxidative stress in endothelial cells without compromising mitochondrial function, supporting previous literature that highlights Boron's ability to neutralize ROS and enhance cellular antioxidant capacity [22–25].

Furthermore, examination of mitochondrial fluorescence in MEF and HUVEC cells shows that DeepA-I promotes biogenesis or mitochondrial function. MitoSPY labeling revealed a substantial increase in mitochondrial fluorescence intensity in MEF cells, suggesting that there was an increase in mitochondrial activity or content. Similarly, HUVEC cells showed decreased ROS levels and increased mitochondrial fluorescence, indicating that DeepA-I promotes mitochondrial activity while reducing oxidative stress. All of these findings suggest that DeepA-I promotes mitochondrial integrity, which is crucial for energy metabolism and redox equilibrium, in addition to acting as an antioxidant. These results support the idea that boron supplementation can enhance mitochondrial health and function, building on earlier research that demonstrated Boron's ability to preserve mitochondrial membrane potential provides mitochondrial protection, reduces oxidative stress, and increase cell survival.

Furthermore, wound healing is a complex and tightly regulated process that involves the coordinated action of immune cells, cytokines, growth factors, and ECM remodelling. Oxidative stress is a key barrier to effective wound healing, and Boron's antioxidant and anti-inflammatory properties are thought to promote tissue regeneration by restoring redox balance and modulating critical cellular processes [13, 16, 26, 27]. In our study, the scratch assay demonstrated that DeepA-I supplementation accelerated wound closure in both cell types. Specifically, HUVEC cells treated with DeepA-I at a 1:1000 ratio exhibited complete wound closure within 48 h, while a higher dose (1:10) further enhanced the healing process. In MEF cells, wound closure was achieved within 24 h under all experimental conditions (Figures 4 and 5). These observations suggest that DeepA-I effectively supports cell migration and proliferation, essential components of wound repair, possibly through its redox-modulating activity.

Crucially, in vivo data showing Boron's advantageous effects on wound healing and oxidative stress management corroborate our in vitro findings. Boron was given orally, topically, and in combination to adult Wistar rats in a study by Konca and Korkmaz [28]. This study demonstrated that Boron treatment significantly enhanced wound healing characteristics, particularly when administered locally and in combination. Furthermore, oxidative stress indicators in the wound tissue were significantly decreased by these therapies. Additionally, Alarslan and Sarıtaş [29] recently examined the function of Boron in intestinal incisional wound healing in rats in an in vivo investigation. They demonstrated that Boron facilitated the healing of wounds resulting from intestinal incisions. The antioxidant and tissue-healing benefits observed in our own research are well supported by these in vivo investigations.

To further evaluate the molecular mechanism of the DeepA-I, we analysed the expression of key proteins involved in cell survival, apoptosis, and oxidative stress response, including Akt, Caspase-3, Nrf2, and PARP (Figure 5). Akt is a central regulator of cell growth, metabolism, and survival [30]. DeepA-I treatment led to differential modulation of Akt protein levels: an increase in HUVEC cells, consistent with enhanced proliferation and wound healing, and a decrease in MEF cells, potentially indicating a cell-type-specific regulatory effect. Interestingly, the expression of Caspase-3, a key effector of apoptosis [31], remained unchanged in MEF cells; however, it increased at the highest DeepA-I concentration (1:10) in HUVEC cells. This suggests a dose-dependent pro-apoptotic effect in endothelial cells at higher Boron concentrations.

Furthermore, DeepA-I treatment increased Nrf2 and PARP protein levels in MEF cells (Figure 5(b)). Nrf2 is a master transcriptional regulator of antioxidant defense genes, playing a critical role in cellular protection against oxidative stress [32]. PARP is essential for DNA repair and maintaining genomic integrity in response to oxidative damage [13]. The upregulation of Nrf2 and PARP in MEF cells suggests that DeepA-I enhances cellular defence mechanisms by activating antioxidant and DNA repair pathways, contributing to overall cellular resilience and stress adaptation.

It has been documented that Boron increases the activation of NRF2, a crucial transcription factor that regulates cellular antioxidant responses, typically through upstream kinases, including Akt, mitogen-activated protein kinases (MAPKs), and protein kinase C (PKC) [33, 34]. After activation, NRF2 binds to the antioxidant response elements (ARE) of target genes. It increases the expression of antioxidant enzymes, including glutathione peroxidase (GPx), superoxide dismutase (SOD), and heme oxygenase-1 (HO-1) [35, 36]. By lowering the ROS level, these enzymes preserve the integrity and functionality of the mitochondria. The activation of NRF2, which increases the expression of antioxidant enzymes, controls mitochondrial activity, and facilitates DNA repair, may account for Boron's regenerative and antioxidant effects. Further research on these mechanisms will provide a better understanding of Boron's potential for tissue repair.

Our data demonstrate that DeepA-I supplementation exerts multifaceted biological effects, including reducing oxidative stress, supporting wound healing through enhanced cell migration and proliferation, and modulation of key molecular pathways involved in cell survival and stress response. Importantly, these effects appear to be cell-type dependent and dose sensitive, highlighting the need for further studies to optimize Boron-based interventions for therapeutic use.

The application of substances with antioxidant, antibacterial, and mitochondrial modulatory qualities is emphasised in recent developments in tissue restoration. Hydrogels, growth factors, and biological dressings that encourage tissue regeneration are examples of standard applications. By altering inflammatory pathways, Boron-based hydrogels, including the guar gum/polyvinyl alcohol/borax-tannic acid (GPBT) hydrogel, have been shown to accelerate the production of granulation tissue and re-epithelialization [37]. Similar to this, bioactive glycerolhydrogels containing silicon and boron aid in tissue repair and infection management by exhibiting broad-spectrum antibacterial action against pathogens frequently present in chronic wounds [38]. Negative Pressure Wound Therapy (NPWT) is often used in conjunction with advanced dressings to enhance wound closure by promoting the production of granulation tissue and reducing edema [28]. Additionally, by improving mitochondrial function and metabolic activity in wound tissues, photobiomodulation (PBM) and oxygen-based therapies aid in the healing process [39]. By enhancing mitochondrial activity and antioxidant defense while simultaneously encouraging cell migration and survival, DeepA-I stands out as a promising multifunctional drug in wound care.

Future studies should focus on in vivo validation of DeepA-I's efficacy and long-term safety profile, as well as exploring the molecular pathways mediating its context-specific effects on epithelial and endothelial cells.

## 5. Conclusion

This study investigated the effects of DeepA-I, a supplement containing boron, on energy metabolism and wound healing in HUVEC and MEF cells. As a result, DeepA-I triggered wound healing by increasing cell migration and reducing ROS levels without causing changes in mitochondrial activity. At the same time, it was observed that DeepA-I, when applied at different doses, did not have a toxic effect on endothelial and epithelial cells. These findings suggest that DeepA-I accelerates wound healing without causing harmful effects and can reduce the adverse effects caused by oxidative stress, especially in the circulatory, respiratory and nervous systems, by reducing ROS, which causes damage to macromolecules through effects such as denaturation, peroxidation, etc. and contributes to the development of many free radical-mediated diseases.

## Figures and Tables

**Table 1:** Concentrations of minerals found in the DeepA-I solution, including Boron.

	Mineral concentrations (mg kg^−1^)					
	B	Ca	K	Mg	Na	S
DeepA-I	111 ± 1	4.2 ± 0.3	7.0 ± 0.7	0.62 ± 0.01	1408 ± 16	4.6 ± 0.1

## Data Availability

Data will be made available on request.

## Supporting Information

Supporting Figure 1. Wound closure (%) of HUVEC (Upper) and MEF (Bottom) cell lines following DeepA-I treatment for 24 and 48 h.
