# Supplementary material for: Boron-Enhanced Mitochondrial Repair: DeepA-I Tissue Regeneration
Source: Scientifica (Cairo). 2025 Oct 26;2025:5343930. doi: 10.1155/sci5/5343930 (PMC12580037; doi:10.1155/sci5/5343930)
Supplement: Supporting Information — Additional supporting information can be found online in the Supporting Information section. [file 5343930.f1.docx]

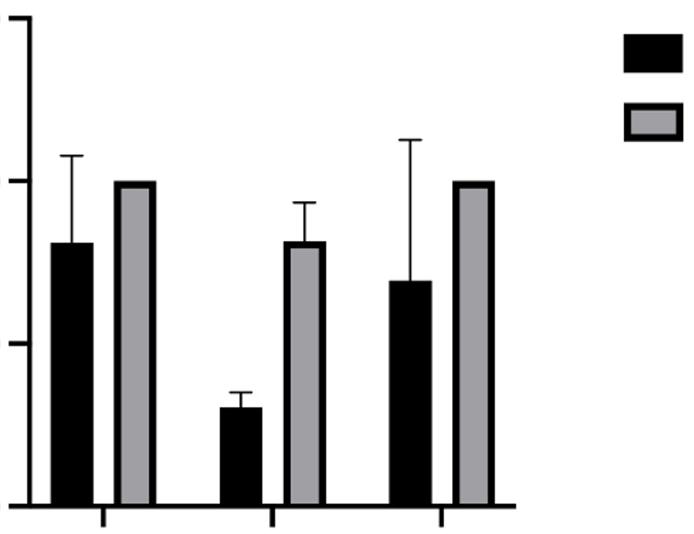


24h

**0**

**50**

**150**

**100**

48h

**% Closure**

**1:1000**

**1:10**

**Control**

**Dosage (µl/ml)**


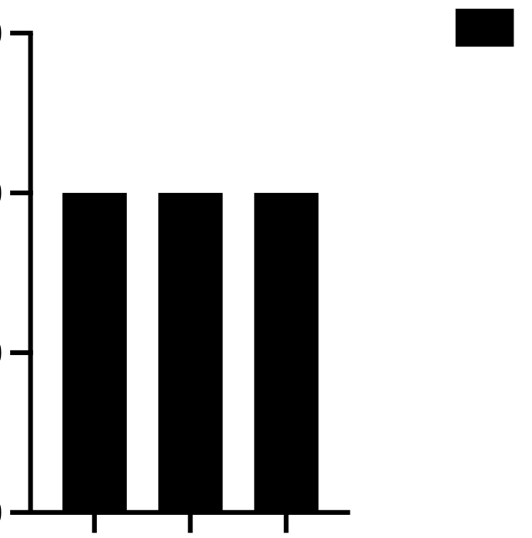


24h

**150**

**100**

**% Closure**

**50**

**0**

**1:1000**

**1:10**

**Control**

**Dosage (µl/ml)**

Supplemental Figure 1. Wound closure (%) of HUVEC (Upper) and MEF (Bottom) cell lines following DeepA-I treatment for 24 h and 48 h.
